# Understanding the Aesthetic and Emotional Impact of Masseter Muscle Prominence: A Pilot Cross‐Sectional Survey Study

**DOI:** 10.1111/jocd.71087

**Published:** 2026-07-22

**Authors:** Alexander Rivkin, Amir Moradi, Julia K. Garcia, Catherine Foley, Annaliza Dominguez

**Affiliations:** ^1^ Rivkin Aesthetics Los Angeles California USA; ^2^ Moradi MD Vista California USA; ^3^ AbbVie Irvine California USA

**Keywords:** burden of disease, emotion, lower facial shape, patient satisfaction, quality of life, signs and symptoms

## Abstract

**Background:**

Masseter muscle prominence (MMP) refers to the visually apparent enlargement of the masseter muscles. This square or trapezoidal lower facial shape can be perceived as aesthetically unappealing. Consequentially, individuals may experience negative emotional and social impacts. Limited research exists characterizing the burden of MMP by severity.

**Aims:**

To evaluate the burden of MMP through a cross‐sectional survey among aesthetically inclined adults.

**Methods:**

Recent recipients of minimally invasive facial or neck aesthetic treatments were invited to complete an online survey assessing appearance‐related impacts, satisfaction, and emotional perceptions of MMP. Responses were stratified by self‐assessed MMP severity. Descriptive statistics, overall group effects, and pairwise odds ratios (ORs) were computed.

**Results:**

A total of 110 participants (95% female; 89% White) completed the survey, with 29% reporting not at all/mildly visible masseter muscles (group A), 31% reporting moderately visible masseter muscles (group B), and 40% reporting visible/very visible masseter muscles (group C). Compared with group A, group C had significantly higher odds of reporting appearance‐related impacts of MMP: lateral jaw protrusion, square or wide lower face (ORs = 37.8, 4.4, and 3.1, respectively; all *p* < 0.05), and emotional impacts of MMP: feeling less attractive and bothered by their lower facial appearance (ORs = 4.2 and 3.4, respectively; all *p* < 0.05). Trends toward dissatisfaction with the lower face and sadness were also observed.

**Conclusions:**

Greater MMP severity was associated with appearance‐related and emotional burden. These real‐world survey findings can increase awareness of MMP burden from the patient perspective and support shared decision‐making between patients and aesthetic providers when discussing treatments.

## 1 Introduction

The masseters are large, paired muscles originating at the zygomatic arch and inserting at the mandibular angle [1]. Aesthetically, they contribute to the contour of the lower face. Functionally, they provide the main force for mouth closure and chewing [1, 2]. Masseter muscle prominence (MMP) refers to the unilateral or bilateral enlargement of the masseter muscles, which can occur either in the absence of pathological findings on physical examination or in association with repetitive jaw activity, such as chewing, clenching, or grinding teeth [2]. Aesthetically, MMP can cause the lower third of the face to appear wide, angular, square shaped, or asymmetric, which may be perceived as unattractive [2, 3]. Affected individuals may pursue treatment to reduce the bulk of the masseter muscles in order to achieve a narrower and more aesthetically pleasing lower face [2, 4, 5]. This would have the desired effect of changing the overall facial shape from square or trapezoidal to ovoid.

Individuals have reported emotional, social, relationship, and work impacts in relation to MMP as part of 2 qualitative research studies aimed at understanding the perspective of individuals with MMP [6, 7]. Emotional impacts were notably among the most frequent spontaneously reported consequences of having MMP in these qualitative interviews [6]. Participants expressed feelings of self‐consciousness as well as decreased attractiveness, femininity, and confidence, and an unwillingness to be photographed as a result of their condition [6, 7]. The impact of MMP also extends to self‐perceived negative social impressions and interactions. Individuals have reported that their MMP makes them appear to be angry or aggressive and scary, which may be detrimental in professional settings. For example, MMP can cause individuals to appear upset to colleagues, which may hinder professional relationships and limit career opportunities as a result [6]. Moreover, individuals also reported that their MMP contributes to feeling shy and receiving negative comments from others [6].

Although these 2 existing qualitative studies have elucidated patient‐reported burdens associated with MMP, sample sizes were small and further research is needed to build on this evidence [6, 7]. The degree to which this burden differs between individuals with varying levels of MMP severity has not been well characterized. A better understanding of this burden from the patient perspective would enable aesthetic providers to proactively discuss this condition and its associated impacts with patients as well as facilitate discussions around potential treatment options to help alleviate the burden. This study evaluated MMP burden in an aesthetically inclined population using a cross‐sectional survey to assess appearance‐ and physical‐related impacts, satisfaction/dissatisfaction with the lower face, and emotional perceptions, such as feeling less attractive and more bothered, related to varying levels of MMP severity.

## 2 Methods

### 2.1 Study Design

**FIGURE 1:**
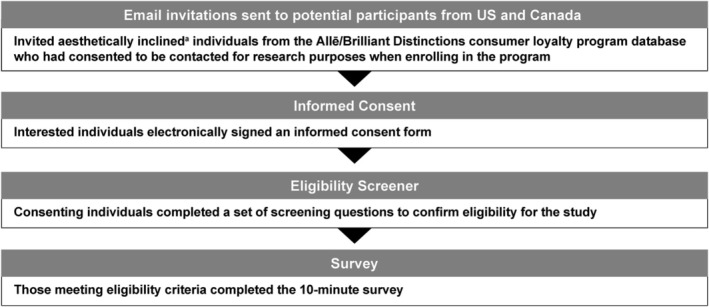
Study design. ^a^Had received at least 1 minimally invasive treatment in the face or neck with Botox, Juvéderm, or Kybella/Belkyra in the past 12 months.

This pilot study evaluated the aesthetic and emotional burden of MMP in aesthetically inclined individuals using a cross‐sectional, online, Health Insurance Portability and Accountability Act (HIPAA)‐compliant survey of participants enrolled in Allergan Aesthetics' consumer loyalty program for aesthetic products (Figure 1). The 10‐min survey was fielded from December 5, 2021, through February 8, 2022. A target sample size of 100 participants was predetermined, and target recruitment quotas were predefined for each MMP severity category, as measured by self‐assessed masseter muscle visibility. Target recruitment quotas were predefined for each MMP severity category to ensure representation across severity groups.

This study was approved by the Western Institutional Review Board‐Copernicus Group (WCG) Institutional Review Board (#00000533; Puyallup, Washington) for both United States (US) and Canada and conducted in compliance with all applicable guidelines for the protection of human participants for research as outlined in 21 Code of Federal Regulations (CFR) 50 and the accepted standards for Good Clinical Practice and International Conference on Harmonization. All participants provided informed consent electronically prior to study enrollment. Participants were compensated with a $10 cash card for completion of the survey.

Individuals were invited to participate in the study if enrolled in the Allē (US) or Brilliant Distinctions (Canada) consumer loyalty program and consented to be contacted for research purposes upon enrollment. In addition, individuals must have been ≥ 18 years old and had to have been aesthetically inclined, meaning they had received at least 1 minimally invasive treatment in the face or neck with Botox, Juvéderm, or Kybella/Belkyra in the past 12 months per the program database to be invited. Individuals received email invitations containing a link to the survey and up to 2 email reminders to participate in the study.

Prior to completing the survey, participants were asked to confirm they had the ability to read and understand English, were currently living in the US or Canada, had a calculated body mass index ≤ 30 kg/m^*2*^ by providing height and weight, were able to locate the masseter muscles by confirming feeling firmness on each side of their lower face (where the back teeth meet) when clenching their teeth, and were able to self‐assess the severity of their MMP. To assist with this self‐assessment, participants were provided a diagram highlighting the location of the masseter muscles and were asked to use a mirror or forward‐facing camera to look at their lower face. MMP severity was measured on a 5‐point descriptor scale ranging from *Not at all visible* to *Very visible*. Participants were excluded if they reported having any feature(s) that would interfere with the assessment of the appearance of the lower face (e.g., a beard, lower facial fat, loose/lax skin, facial trauma), muscle paralysis or weakness in the lower face, or prior treatment with any facial‐shaping procedure in the lower face within the previous 30 days (e.g., botulinum toxin for MMP, fillers, skin‐tightening lasers, fat‐reducing injectables).

Most survey questions were adapted from patient‐reported outcome (PRO) measures that were developed and validated by Allergan Aesthetics according to the US Food and Drug Administration's Patient‐Reported Outcome Measures guidance [6, 7, 8]. The survey included demographic questions, 4 items assessing appearance‐related impacts of MMP (jaw protruding laterally, square, uneven, and wide lower face), 1 item assessing teeth clenching frequency (a sign of potential bruxism) over the past 24 h, and 1 item assessing satisfaction/dissatisfaction with the appearance of their lower face. In addition, 6 items assessing emotional perceptions from MMP over the past week included feeling bothered, less attractive, self‐conscious, less confident, sad, and embarrassed.

### 2.2 Statistical Analysis

Survey responses and participant demographic data were analyzed descriptively using frequencies, percentages, and means, and were stratified into 3 groups based on self‐assessed level of MMP severity. Group A included participants reporting *Not at all visible* or *Mildly visible* masseters, group B included *Moderately visible* masseters, and group C included participants with *Visible* or *Very visible* masseters. *p* values were computed from the overall Wald chi‐square test via unadjusted logistic regression models to determine whether an overall group effect was statistically significant between groups A, B, and C. If the overall group effect was statistically significant at *p* ≤ 0.05, then pairwise odds ratios (ORs) were computed using group A as a reference group (i.e., group B vs. A and group C vs. A).

## 3 Results

**FIGURE 2:**
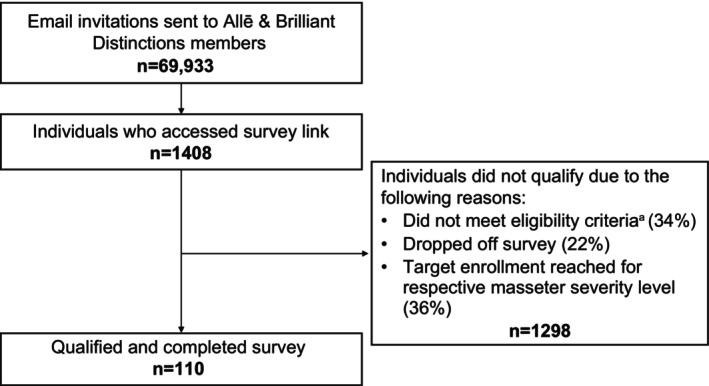
Participant disposition. ^a^Reasons for ineligibility: 26% did not consent, 9% restarted screener, 23% received recent facial‐shaping procedure, 15% had no masseter muscle firmness, 10% had BMI > 30 kg/m^2^, 20% opted out of providing height/weight, 6% had feature or condition in lower face, 1% had paralysis in lower face. BMI, body mass index.

**FIGURE 3:**
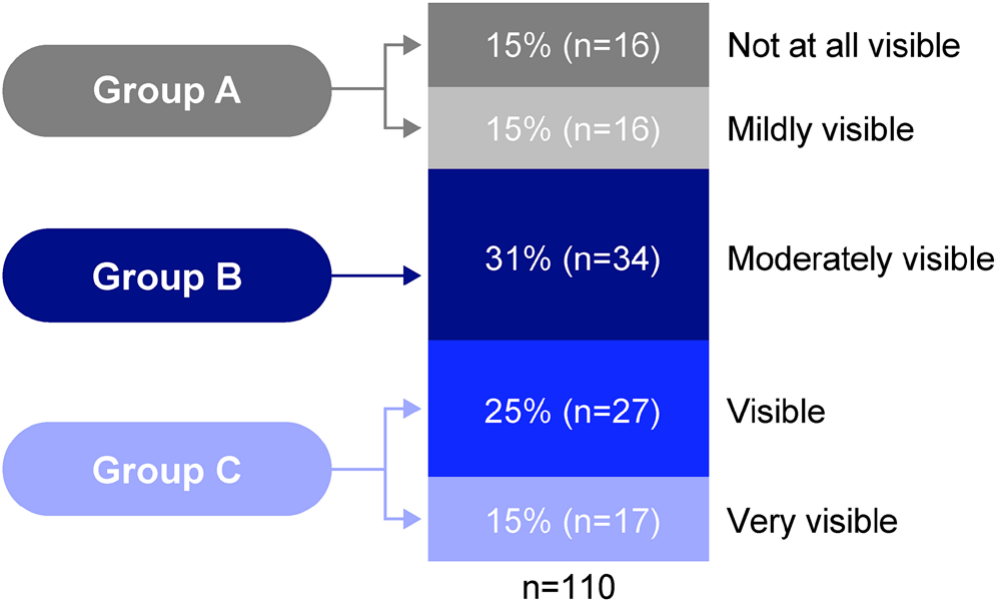
Masseter visibility groups for analysis. All outcomes were analyzed by 3‐group stratification depending on self‐reported masseter visibility. Total percentage is above 100 due to rounding of each visibility group.

A total of 68 933 email invitations to participate in the online survey were sent to both Allē and Brilliant Distinctions members, and 1408 of those invited clicked on the survey link provided within the invitation (Figure 2). A total of 110 participants qualified and completed the pilot survey, reaching target enrollment for the study. The majority of respondents were female (95%), White (89%), and had a mean (SD) age of 48.7 (12.1) years. Twenty‐nine percent of respondents reported that their masseter muscles were *Not at all visible* or *Mildly visible* (group A), 31% reported *Moderately visible* masseter muscles (group B), and 40% reported *Visible* or *Very visible* masseter muscles (group C) (Table 1, Figure 3). There were no statistically significant demographic differences in country of residence, gender identity, age, body mass index (BMI), household income, or highest education level observed across visibility groups, as determined from overall Wald chi‐square test in ordinal regression (Table 1). Most respondents were naive to aesthetic MMP treatment; those with greater MMP severity had 4.2 times higher odds of receiving past MMP treatment (group C vs. group A: OR = 4.2; *p* = 0.02; Table 2).

**TABLE 1:** Participant‐reported demographic and baseline characteristics by masseter visibility groups.

	Total (*N* = 110)	Group A (Not at all/Mildly visible) (*n* = 32)	Group B (Moderately visible) (*n* = 34)	Group C (Visible/Very visible) (*n* = 44)
Country, *n* (%)				
US	66 (60)	16 (50)	18 (53)	32 (73)
Canada	44 (40)	16 (50)	16 (47)	12 (27)
Gender identity, *n* (%)				
Female	105 (95)	30 (94)	34 (100)	41 (93)
Male	4 (4)	1 (3)	0 (0)	3 (7)
Prefer not to answer	1 (1)	1 (3)	0 (0)	0 (0)
Age				
Mean, years (SD)^a^	48.7 (12.1)	49.2 (11.1)	50.7 (14.0)	46.8 (11.2)
Prefer not to answer, *n* (%)	4 (4)	1 (3)	2 (6)	1 (2)
BMI				
Mean, kg/m^2^ (SD)	22.3 (3.3)	21.9 (2.3)	22.6 (3.1)	22.3 (4.0)
Race/Ethnicity^b^, *n* (%)				
Hispanic	4 (4)	1 (3)	0 (0)	3 (7)
Black or African American	0 (0)	0 (0)	0 (0)	0 (0)
Asian or Pacific Islander	3 (3)	1 (3)	1 (3)	1 (2)
Native American Indian	0 (0)	0 (0)	0 (0)	0 (0)
Caucasian or White	98 (89)	29 (91)	31 (91)	38 (86)
Other	1 (1)	0 (0)	0 (0)	1 (2)
Prefer not to answer	4 (4)	1 (3)	2 (6)	1 (2)
Annual household income, *n* (%)				
<$75 000	14 (13)	1 (3)	6 (18)	7 (17)
≥ $75 000	75 (68)	25 (78)	21 (62)	29 (66)
Prefer not to answer	21 (19)	6 (19)	7 (21)	8 (18)
Highest level of education, *n* (%)				
High school graduate	10 (9)	4 (12)	2 (6)	4 (9)
Some college/graduated college	61 (56)	15 (47)	19 (56)	27 (61)
Some post‐graduate studies/graduated with post‐graduate degree	34 (30)	12 (37)	9 (27)	13 (29)
Prefer not to answer	5 (5)	1 (3)	4 (12)	0
History of aesthetic treatment for MMP, *n* (%)				
Never received treatment	80 (73)	27 (84)	26 (76)	27 (61)
Received treatment once	10 (9)	1 (3)	3 (9)	6 (14)
Received treatment more than once	18 (16)	4 (12)	3 (9)	11 (25)
Prefer not to answer	2 (2)	0 (0)	2 (6)	0 (0)

*Note:* With exception to history of aesthetic treatment for MMP, there were no significant differences observed across visibility groups for other demographic variables, as determined from the overall Wald chi‐square test in ordinal regression.

Abbreviations: BMI, body mass index; MMP, masseter muscle prominence; SD, standard deviation.

^a^ Mean represents *n* = 106 participants who reported their age.

^b^ Participants were asked to “select all that apply.”

**TABLE 2:** Overall group effect observations and pairwise odds ratio compared with n*ot at all visible/*m*ildly visible* Masseter Muscle Group.

Survey Item	*p* ^a^	Pairwise OR (95% CI)^b^	
		Group B vs Group A	Group C vs Group A
History of aesthetic treatment for MMP, *n* (%)			
Never received treatment	0.02	0.57 (0.17, 1.89)	0.24 (0.08, 0.69)
Appearance‐related impacts of MMP			
Jaw protruded laterally	< 0.01	8.28 (2.85, 24.10)	37.77 (11.97, 119.19)
Square jaw	< 0.01	1.53 (0.61, 3.83)	4.44 (1.81, 10.87)
Wide jaw	0.03	2.86 (1.13, 7.22)	3.09 (1.28, 7.43)
Unevenness	0.44	—	—
Teeth clenching frequency	< 0.01	3.16 (1.23, 8.12)	4.85 (1.96, 11.99)
Satisfaction with lower face appearance	0.07	—	—
Emotional impact			
Bothered	< 0.01	3.09 (1.19, 8.00)	4.19 (1.67, 10.52)
Less attractive	0.03	2.48 (0.97, 6.37)	3.37 (1.37, 8.29)
Sad	0.07	—	—
Self‐conscious	0.21	—	—
Embarrassed	0.53	—	—
Less confident	0.11		

Abbreviations: CI, confidence interval; MMP, masseter muscle prominence; OR, odds ratio.

^a^ *p* ≤ 0.05 indicates an overall group effect (determined via Wald chi‐square tests in ordinal logistic regression models) between masseter visibility groups: group A (*Not at all visible*/*Mildly visible*) vs. group B (*Moderately visible*) vs. group C (*Visible*/*Very visible*).

^b^ Pairwise odds ratios were computed for group B vs. A and group C vs. A only if the *p* value of overall group effect was ≤ 0.05.

**FIGURE 4:**
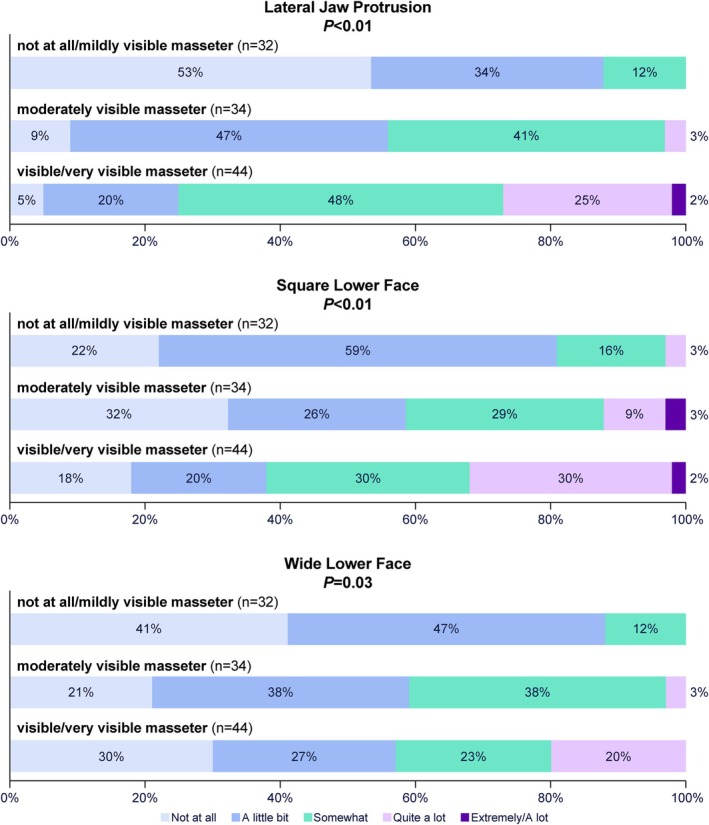
Participant‐reported appearance‐related impacts of MMP. MMP, masseter muscle prominence.

**FIGURE 5:**
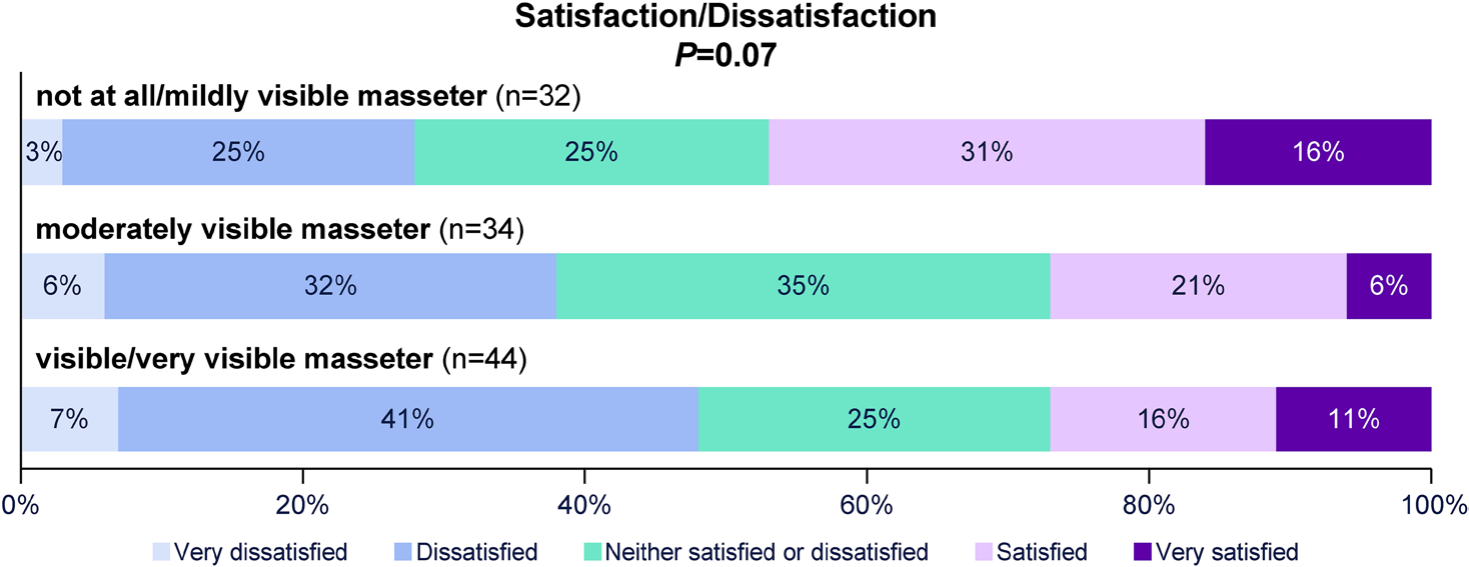
Participant‐reported satisfaction/dissatisfaction with lower facial appearance.

**FIGURE 6:**
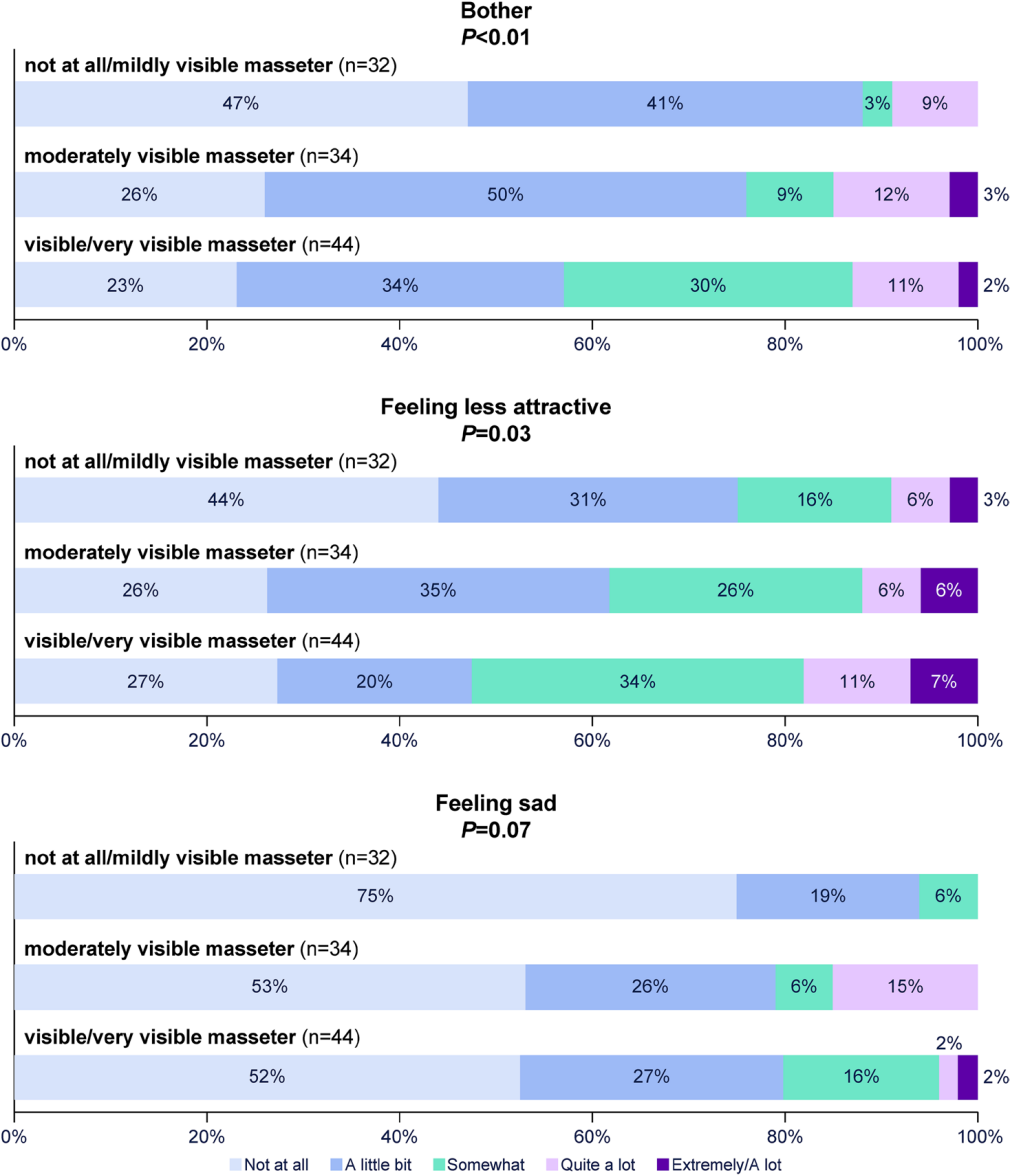
Participant‐reported emotional perceptions of MMP. MMP, masseter muscle prominence.

Compared with participants who had less severe MMP (group A), participants with greater MMP severity (group C) had higher odds of perceiving appearance‐related impacts of MMP, including that their jaw protruded laterally (OR = 37.8, *p* < 0.01), their lower face was square (OR = 4.4, *p* = 0.01), and their lower face looked wide (OR = 3.1, *p* = 0.03; Figure 4, Table 2). No statistically significant group differences were found for reports of lower face unevenness. Compared with group A, group C had 4.9 times higher odds of frequent teeth clenching (OR = 4.9; *p* < 0.01; Table 2). There was also a trend toward dissatisfaction with the lower face in those with greater masseter visibility compared with those with less visibility; however, no statistically significant group differences were observed (Figure 5, Table 2). Further, in terms of emotional impacts, compared with group A, group C had higher odds of feeling bothered and less attractive (ORs = 4.2 and 3.4, respectively; *p* ≤ 0.03) (Figure 6, Table 2). Generally, there was a trend toward participants with greater MMP severity feeling sad, self‐conscious, embarrassed, and less confident compared with participants who had less severe MMP, which did not reach statistical significance.

## 4 Discussion

In past qualitative studies, individuals with MMP have reported various emotional and social impacts associated with their condition, which can interfere with their overall well‐being [3, 6]. Negative impacts can motivate individuals to seek treatment for their condition to relieve burden and improve quality of life [3]. The present study builds on the previous qualitative research by demonstrating that participants who perceive themselves as having greater MMP severity have higher odds of reporting clenching their teeth, being bothered by their lower facial appearance, and feeling less attractive. In addition, individuals tend to be less satisfied with their lower facial appearance and feel sadder than those with less severe MMP. A deeper understanding of the impact of MMP, as highlighted through this research, can enable providers to proactively consult patients on the burden attributed to their condition and raise awareness on potential treatment options.

Survey questions in the present study were adapted from PRO measures developed from previous qualitative research, which demonstrated patients with MMP viewed concepts related to appearance, symptoms, emotional impact, and satisfaction as important [6]. The study results reported herein corroborate findings of past research through participant assessment of similar factors (e.g., facial shape, teeth clenching, satisfaction, and feeling self‐conscious, less confident, sad, and embarrassed). Additionally, the study results confirm the importance of these concepts in individuals with MMP and demonstrate that the magnitude of participant concern for each factor increased with greater MMP severity. Despite a large proportion of participants reporting greater perceived MMP severity, most had not received prior treatment, which may reflect limited awareness of MMP as a treatable aesthetic concern or a lack of patient prioritization for this particular area. Results from this study also align with observations from another qualitative study focused on developing MMP PRO measures in the Asia‐Pacific region [7]. Similar to the present study, the most frequently spontaneously reported signs and symptoms of MMP in the Asia‐Pacific regional study included having a prominent jaw, square shape face, and teeth clenching. Feeling less attractive was among the top 3 spontaneously reported impacts by participants in the Asia‐Pacific region, which was observed to be a statistically significant factor in our study when comparing between masseter visibility groups. Furthermore, our study extends and adds granularity to both previous qualitative studies by confirming that the severity of emotional perceptions is largely driven by the severity of a participant's masseter muscle.

A notable strength of this research is the inclusion of a representative sample of aesthetically inclined adults who had recently invested out‐of‐pocket in an aesthetic injectable treatment. Survey responses from this sample provide a snapshot of real‐world impacts of MMP and enhance the validity of findings outside of a standardized clinical study setting. In addition, items used to assess MMP‐related burden in this cross‐sectional survey were adapted from PRO measures developed and validated according to global regulatory standards, which reinforce the relevancy of asking about specific appearance, physical, and emotional concepts that are important to patients with the condition.

A possible limitation of this study is that MMP severity levels were self‐assessed without objective clinical evaluation, which may introduce variability and potential misclassification. To provide guidance with their self‐assessment, participants were screened for being able to locate and palpate the masseter muscles and were provided with a diagram highlighting the location of the masseter muscles for reference. As previously mentioned, while self‐assessment of MMP severity reflects unique, real‐world assessments from participants outside of a clinical setting and represents their own perceptions about their lower face, validated clinician‐reported severity assessments are considered the gold standard and could be considered for implementation in future larger‐scale studies.

Another limitation is that the majority of participants were White females sampled from Allergan Aesthetics' consumer loyalty database. Target sample size was predetermined based on practical considerations rather than a formal power analysis. Recruitment quotas were predefined across MMP severity categories, so the distribution of severity observed in this study is influenced by the study design and should not be interpreted as reflecting prevalence in the general population. Additionally, the logistic regression models were unadjusted, and the study did not employ matching or adjustment for potential confounding variables. In turn, generalizability of results of this pilot sample to other populations may be limited. Results of this study are aligned with the findings from the qualitative research among participants in the Asia‐Pacific region, despite differences in demographics between both populations. Nonetheless, to confirm results, future studies with larger samples should aim to include more ethnically and gender‐diverse populations for global representation, especially in Asian countries where MMP treatment is prevalent. Given the perception‐based nature of these data, findings should be interpreted in the context of the study design and not as objective clinical assessments.

## 5 Conclusions

This real‐world study reveals that individuals with greater MMP severity are inclined toward experiencing negative appearance‐related impacts of MMP, such as lateral jaw protrusion and a square lower face, as well as dissatisfaction, bother, and negative emotional perceptions toward the appearance of their lower face. These findings align with previous qualitative studies on MMP and confirm that the concepts assessed, such as feeling less attractive, self‐conscious, sad, and embarrassed, are relevant and important from the patient perspective. Improved understanding of patient perception may support more informed discussion between patients and physicians.

## Author Contributions

All authors participated in the study design, research, analysis, and drafting of the manuscript, and gave approval for the study.

## Funding

Allergan Aesthetics, an AbbVie company, funded this study and participated in the study design, research, analysis, data collection, interpretation of data, reviewing, and approval of the publication.

## Ethics Statement

This study was approved by the Western Institutional Review Board‐Copernicus Group (WCG) Institutional Review Board (#00000533; Puyallup, Washington) for both United States (US) and Canada and conducted in compliance with all applicable guidelines for the protection of human participants for research as outlined in 21 Code of Federal Regulations (CFR) 50 and the accepted standards for Good Clinical Practice and International Conference on Harmonization.

## Conflicts of Interest

Alexander Rivkin: is an investigator and consultant for Galderma, Merz, Suneva, and Allergan Aesthetics, an AbbVie company. Amir Moradi: is an investigator and consultant for Allergan Aesthetics, an AbbVie company. Julia K. Garcia: is a full‐time employee of AbbVie and may own AbbVie stock. Catherine Foley: is a full‐time employee of AbbVie and may own AbbVie stock. Annaliza Dominguez: is a full‐time contractor with AbbVie and may own AbbVie stock. The opinions expressed in this article are those of the authors. All authors had access to relevant data and participated in the drafting, review, and approval of this publication. No honoraria or payments were made for authorship.

## Data Availability

AbbVie is committed to responsible data sharing regarding the clinical trials we sponsor. This includes access to anonymized, individual, and trial‐level data (analysis data sets), as well as other information (e.g., protocols, clinical study reports, synopses, or statistical analysis plans), as long as the trials are not part of an ongoing or planned regulatory submission. These clinical trial data can be requested by any qualified researchers who engage in rigorous, independent, scientific research, and will be provided following review and approval of a research proposal, Statistical Analysis Plan (SAP), and execution of a Data Use Agreement (DUA). Data requests can be submitted at any time after approval in the US and Europe and after acceptance of this manuscript for publication. The data will be accessible for 12 months, with possible extensions considered. For more information on the process or to submit a request, visit the following link: https://vivli.org/ourmember/abbvie/ then select “Home.”
